# Genetic evidence suggests that depression increases the risk of erectile dysfunction: A Mendelian randomization study

**DOI:** 10.3389/fgene.2022.1026227

**Published:** 2022-10-14

**Authors:** Kai Ma, Pan Song, Zhenghuan Liu, Luchen Yang, Linchun Wang, Jing Zhou, Junhao Chen, Qiang Dong

**Affiliations:** Department of Urology, Institution of Urology, West China Hospital of Sichuan University, Chengdu, Sichuan, China

**Keywords:** depression, erectile dysfunction, causal estimates, Mendelian randomization, single-nucleotide polymorphisms

## Abstract

**Background:** The causal relationship between depression and erectile dysfunction (ED) is still uncertain.

**Objectives:** To identify the genetically predicted causality of depression on ED through Mendelian randomization (MR).

**Materials and methods:** A comprehensive GWAS meta-analysis comprising 807,553 Europeans provided single-nucleotide polymorphism (SNP) information for depression, and another genome-wide association analysis involving 223,805 European ancestries measured SNPs for ED. The inverse variance weighted (IVW) method was used as the primary MR analysis method to evaluate causal effects. In addition, the maximum likelihood method, MR-Egger, weighted median, robust adjusted contour score (MR.RAPS), and MR pleiotropic residual and outlier (MR-PRESSO) methods were used as supplements for sensitivity analysis.

**Results:** According to the IVW analysis, depression significantly increases the incidence of ED (odds ratio [OR] = 1.68, 95% confidence interval [CI] = 1.38–2.05, *p* < 0.001). In sensitivity analyses, the ORs for the maximum likelihood method, MR-Egger, weighted median, MR.RAPS, and MR-PRESSO are 1.70 (95% CI = 1.39–2.08, *p* < 0 .001), 1.94 (95% CI = 0.63–6.01, *p* > 0 .05), 1.59 (95% CI = 1.21–2.10, *p* < 0 .001), 1 .70 (95% CI = 1.39–2.08, *p* < 0 .001), and 1.68 (95% CI = 1.40–2.04, *p* < 0 .001). There is no clear indication of potential heterogeneity or pleiotropy (*p* for the MR-Egger intercept = 0.804; *p* for the global test = 0.594; and *p* for Cochran’s Q statistics >0.05).

**Conclusion:** Genetically predicted depression plays a potentially causal role in the occurrence of ED.

## Introduction

The inability to generate or sustain an adequate penile erection for satisfying sexual performance is referred to as erectile dysfunction (ED), a traditional male sexual dysfunction that increases with men’s age (NIH Consensus Conference, 1993). As a global problem, it is predicted that there will be 322 million cases of ED by 2025 (Ayta et al., 1999; McKinlay, 2000). The causes of ED are numerous and could be organic (e.g., vascular and neurogenic), psychogenic (e.g., excessive performance anxiety), or mixed (Salonia et al., 2020).

Depression, a common psychiatric disorder, consists of many clinical features, including sensations of melancholy and despair, lack of enthusiasm for enjoyable activities (anhedonia), disruption in appetite, sleep disturbances, fatigue, and difficulty in concentrating (Price, 2004). Clinical depression, excluding bipolar (manic-depressive) illnesses, is typically categorized as a major depressive disorder (MDD) or dysthymia, based on the extent. Both diseases were referred to as depression in this article.

Although depressive illness and ED appeared to have high comorbidity, their causal relationship was still unclear (Seidman and Roose, 2000). There were several proofs suggesting that depression may induce ED. Steiger et al. (1993) reported that the ED symptoms accessed by nocturnal penile tumescence (NPT) in depressed men were diminished after antidepressant treatment. After that, Thase et al. (1988) and Thase et al. (1992) assessed that the NPT time and penile rigidity were significantly impaired in depressed men compared with men without depression. Araujo et al. (1998) demonstrated that those who suffered from depressive symptomatology were 1.82 times more likely to get impotence independent of factors such as income and age. Moreover, some research also disclaimed that the risk of depressive symptoms also elevated among men who presented with moderated or completed erectile dysfunction (Laumann et al., 2008; Takao et al., 2011; Chou et al., 2015). However, these studies were restricted by confounding factors and reverse causation. It was possible to develop both conditions as a result of substance abuse or medical illness, or these conditions might be comorbid simply because they are highly prevalent, especially in older men, regardless of their etiology (Seidman and Roose, 2000).

Mendelian randomization (MR) was an epidemiological study design that could be applied to investigate the potential causality between depression and ED (Smith and Ebrahim, 2003). In Mendelian randomization, single-nucleotide polymorphisms (SNPs) served as proxy indicators of instrumental variables (IVs) of exposure. These genetic variants were used to evaluate exposure’s causal effect on outcome variables at the genetic level. Mendelian randomization has the following desirable features: 1) genetic variant measurements were highly accurate and can capture long-term exposure effects, thus avoiding estimation bias caused by the measurement error (Haycock et al., 2016). 2) The alleles were randomly segregated when forming a zygote and such segregation is independent of postnatal confounders, making the results less vulnerable to potential confounders and reverse causation (Smith and Ebrahim, 2003). Therefore, MR analysis imitated a naturally emerged controlled trial (RCT) to evaluate causal estimates.

Up to now, no study has elucidated the causes of the connections between depression and ED. Hence, using two-sample Mendelian randomization, our study investigated whether depression may be causally related to the onset of ED at the genetic level.

## Materials and methods

### MR assumptions

This study was conducted under the guideline of the reporting MR study, and the STORBE-MR checklist is provided in the Supplementary Material (Skrivankova et al., 2021a). There were three main assumptions needed to be satisfied in the MR study: (A-I) the instrument variables (IVs) are associated with exposure; (A-II) the IVs are not correlated with confounding factors; and (A-III) the IVs influence the outcome only through exposure (Skrivankova et al., 2021a; Skrivankova et al., 2021b).

### Data sources

The GWAS performed by Bovijn et al. (2019), the largest GWAS of ED at present, was obtained as the summary-level dataset of ED. This comprehensive study recruited 223,805 European males (6,175 cases and 217,630 controls) from the hospital-recruited Partners HealthCare Biobank cohort, the United Kingdom Biobank (UKB) cohort, and the Estonian Genome Center of the University of Tartu cohort. ED cases were identified by self-report, doctor-diagnosed, taking oral ED drugs, or surgery history for ED.

For the SNPs of depression, we extracted from the comprehensive GWAS meta-analysis comprising 807,553 Europeans (246,363 cases and 561,190 controls) and consisted of 23andMe_307k, United Kingdom Biobank, and PGC_139k cohorts (Howard et al., 2019). The depression phenotype definition ranged from “broad depression” (self-reported seeking assistance for issues with stress, anxiety, or sadness), self-reported depressive symptoms with accompanying disability and depression diagnosed clinically, and MDD clinically diagnosed. Further detailed information on these two phenotypes was obtained through previous publications (Bovijn et al., 2019; Howard et al., 2019).

### Instrument variable selection

First, 102 single-nucleotide polymorphisms (SNPs) with genome-wide statistical significance ≥ 5 × 10^−8^ of depression were identified by Howard et al. (2019). F-statistics were used to calculate each SNP’s strength using the following formula: $F=R^{2}\times \left(N-2\right)÷\left(1-R^{2}\right)$ , where *R* ^2^ is the proportion of variance explained and N is the total sample size. To calculate *R* ^2^, we used the following formula: 2×EAF×(1−EAF)×Beta ^2^, where EAF is the effect allele frequency and Beta represented the estimated genetic effect on the risk of depression. The total *R* ^2^ explained by the instrumental variables was 0.02. F-statistics > 10 were typically used as the cutoff for powerful IVs (Burgess and Thompson, 2011). In this step, no SNPs were excluded because the F-statistic was above 10 in all of the depression variations. Furthermore, to ensure the IVs were assorted randomly during gestation, SNPs whose *r* ^2^ ≥ 0.001 at a window size of 10,000 Kb for 1,000 Genomics European reference panels were pruned to avoid linkage disequilibrium (LD). Additionally, the SNPs not present in the LD reference panel were also disregarded. Consequently, 24 SNPs were eliminated due to LD, leaving 78 SNPs. In addition, five palindromic SNPs were removed from the retrieved SNPs. The remaining 73 SNPs were used for the subsequent MR analysis. Table 1 shows the specific details of the IVs for depression.

**TABLE 1:** SNPs used as instrumental variables of depression on ED in the MR analyses.

SNP	chr	Position	A1	A2	Frequency A1	Effect size	SE	*p*-value	Sample	R^2^	F-statistic
rs301799	1	8489302	T	C	0.5694	−0.025	0.0035	1.36E−12	807553	3.06E−04	248
rs1002656	1	37192741	T	C	0.7033	−0.0266	0.0038	3.74E−12	807553	2.95E−04	239
rs1466887	1	37709328	T	C	0.5511	−0.0199	0.0036	4.12E−08	807553	1.96E−04	158
rs11579246	1	50559162	A	G	0.9067	0.0381	0.0061	5.71E−10	807553	2.46E−04	198
rs1890946	1	52342427	T	C	0.4671	−0.0235	0.0035	2.68E−11	807553	2.75E−04	222
rs10789214	1	67146817	T	C	0.5661	0.0193	0.0035	4.44E−08	807553	1.83E−04	148
rs2568958	1	72765116	A	G	0.6156	0.0373	0.0036	8.47E−25	807553	6.58E−04	532
rs113188507	1	80809636	A	G	0.2838	0.0221	0.0039	1.87E−08	807553	1.99E−04	160
rs10913112	1	1.76E+08	T	C	0.3767	−0.0264	0.0036	3.40E−13	807553	3.27E−04	264
rs17641524	1	1.98E+08	T	C	0.2091	−0.032	0.0043	1.52E−13	807553	3.39E−04	274
rs1568452	2	58012833	T	C	0.3851	0.0248	0.0036	8.12E−12	807553	2.91E−04	235
rs7585722	2	86819128	T	C	0.8458	−0.0269	0.0048	2.68E−08	807553	1.89E−04	152
rs1226412	2	1.57E+08	T	C	0.7917	0.0256	0.0043	3.46E−09	807553	2.16E−04	175
rs62188629	2	2.08E+08	A	G	0.3136	0.0236	0.0038	7.13E−10	807553	2.40E−04	194
rs4346585	3	44736493	T	C	0.696	−0.0236	0.0038	7.13E−10	807553	2.36E−04	190
rs141954845	3	61192911	A	G	0.388	0.0229	0.0037	8.15E−10	807553	2.49E−04	201
rs6783233	3	1.18E+08	T	C	0.2833	0.0218	0.0039	2.90E−08	807553	1.93E−04	156
rs1095626	3	1.58E+08	T	C	0.5799	−0.0264	0.0035	7.13E−14	807553	3.40E−04	274
rs7685686	4	3207142	A	G	0.5753	0.0202	0.0039	2.57E−08	807553	1.99E−04	161
rs34937911	4	42110353	T	C	0.8838	0.0304	0.0035	4.13E−08	807553	1.90E−04	153
rs45510091	4	1.23E+08	A	C	0.9472	0.0448	0.0055	1.83E−08	807553	2.01E−04	162
rs35553410	4	1.31E+08	T	C	0.7462	−0.0244	0.008	1.42E−09	807553	2.26E−04	182
rs7659414	4	1.77E+08	A	C	0.5782	−0.0201	0.004	1.20E−08	807553	1.97E−04	159
rs60157091	5	61509655	T	G	0.515	0.02	0.0035	1.42E−08	807553	2.00E−04	161
rs3099439	5	87545318	T	G	0.5288	−0.0276	0.0035	5.05E−15	807553	3.80E−04	307
rs10061069	5	93071630	C	C	0.2212	−0.0275	0.0035	8.15E−11	807553	2.61E−04	210
rs30266	5	1.04E+08	A	G	0.3296	0.0308	0.0042	1.45E−16	807553	4.19E−04	339
rs11135349	5	1.65E+08	A	C	0.4713	−0.0295	0.0037	6.04E−17	807553	4.34E−04	350
rs200949	6	27835435	A	C	0.8744	0.048	0.0035	2.53E−19	807553	5.06E−04	409
rs9363467	6	66565703	T	C	0.6035	0.0237	0.0053	6.44E−11	807553	2.69E−04	217
rs725616	6	1.48E+08	T	G	0.3644	0.0204	0.0036	1.87E−08	807553	1.93E−04	156
rs3823624	7	2110346	T	G	0.8067	0.0272	0.0036	1.99E−09	807553	2.31E−04	186
rs2043539	7	12253880	A	G	0.4177	0.0273	0.0045	9.89E−15	807553	3.63E−04	293
rs58104186	7	1.09E+08	A	G	0.4689	0.0237	0.0035	1.82E−11	807553	2.80E−04	226
rs7837935	8	65562019	T	G	0.1522	−0.0292	0.0035	3.34E−09	807553	2.20E−04	178
rs67436663	8	71347626	C	G	0.2402	−0.0259	0.0049	9.37E−10	807553	2.45E−04	198
rs1982277	9	11513019	T	G	0.7594	0.0279	0.0042	1.45E−11	807553	2.84E−04	230
rs3793577	9	23737627	A	C	0.4665	−0.0229	0.0041	8.41E−11	807553	2.61E−04	211
rs59283172	9	25232978	A	G	0.1069	−0.0329	0.0057	1.02E−08	807553	2.07E−04	167
rs703081	9	36999369	T	C	0.3736	0.0253	0.0036	3.07E−12	807553	3.00E−04	242

### Statistical analyses

**FIGURE 1:**
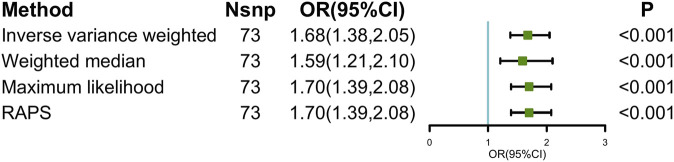
Causal estimates of depression on ED in MR. Abbreviations: ED, erectile dysfunction; MR, Mendelian randomization; Nsnp, number of single-nucleotide polymorphism used in MR analysis; OR, odds ratio; CI, confidence interval; RAPS, robust adjusted profile score.

To obtain MR estimates of depression on erectile dysfunction, we performed the inverse variance weighting (IVW) approach as the main result of MR analysis. For each ${SNP}_{i}$ , the IVW method used the following formula: ${\hat{\beta }}_{Y_{i}}=\theta {\hat{\beta }}_{X_{i}}+\varepsilon_{i},\;\varepsilon_{i}\;∼\;N\left(0,\;se{\left({\hat{\beta }}_{Y_{i}}\right)}^{-2}\right)$ , where ${\hat{\beta }}_{Y_{i}}$ is the effect of ${SNP}_{i}$ on the outcome (erectile dysfunction), ${\hat{\beta }}_{X_{i}}$ represents the effect of ${SNP}_{i}$ on exposure (depression), and θ is the effect of depression on ED. Then, the IVW method combines each SNP effect to an overall weighted effect, generating consistent estimation when all SNPs are valid (Burgess et al., 2013). Other supplemental MR analysis methods were also used to calculate the causal effects, as shown in Figure 1.

We conducted sensitivity analysis mainly under three aspects: the heterogeneity test, the pleiotropy test, and the leave-one-out sensitivity test. The MR-Egger and maximum likelihood approaches were used to test the heterogeneity. Cochran’s Q statistic was utilized to quantify the heterogeneity. In particular, heterogeneity was identified if the Cochran Q test’s *p* value <0.05. Many sensitivity studies were carried out to test and lessen the impact of potential pleiotropy on the results, including the weighted median, MR-Egger, robust adjusted profile score (MR.RAPS), and MR Pleiotropy Residual Sum and Outlier (MR-PRESSO). The weighted median demands that variables from reliable instruments receive 50% of the total weight (Bowden et al., 2016). Even if all SNPs are invalid, the MR-Egger approach can still produce unbiased estimates (Bowden et al., 2015). In addition, the intercept term taken from MR-Egger could be adopted to quantify the directional pleiotropy. MR.RAPS could produce reliable causal estimates by running a linear model while accounting for the profile likelihood of the summary data, even when weak IVs existed (Zhao et al., 2019). In order to find the horizontal pleiotropic outliers, MR-PRESSO (MR-PRESSO) methods were applied. Outlying SNPs were then eliminated, and the effect estimates were re-evaluated (Verbanck et al., 2018). In addition, we used the leave-one-out method to determine which IVs had a significant impact on the estimates. This method utilized the IVW method to repeat MR analysis after excluding IV in turn.

An online tool (https://shiny.cnsgenomics.com/mRnd/) is used to assess the statistical power to identify the difference. The statistical power of depression on ED is 100% when the type I error rate is 0.05 (Brion et al., 2013).

All analyses and figures were made by the packages TwoSampleMR (version 0.5.6), MRPRESSO (version 1.0), and mr.raps (version 0.2) in R (version 4.2.0). *p* < 0.05 (two-sided) was considered statistically significant (Wald, 1940).

## Results

### MR estimates of depression on ED

**FIGURE 2:**
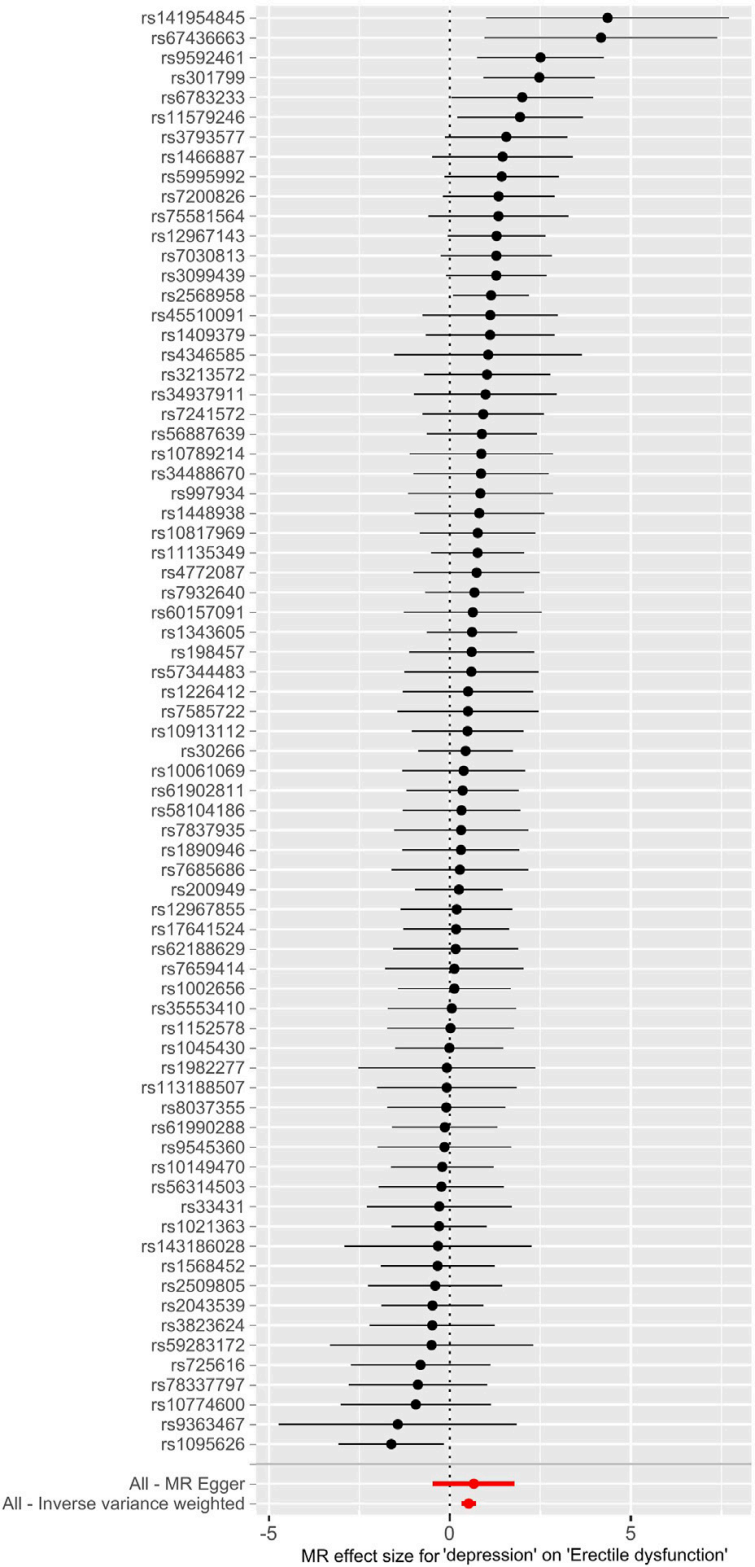
Forest maps of each SNP’s effect for depression on ED and all estimates. Abbreviations: SNP, single-nucleotide polymorphism; ED, erectile dysfunction.

**FIGURE 3:**
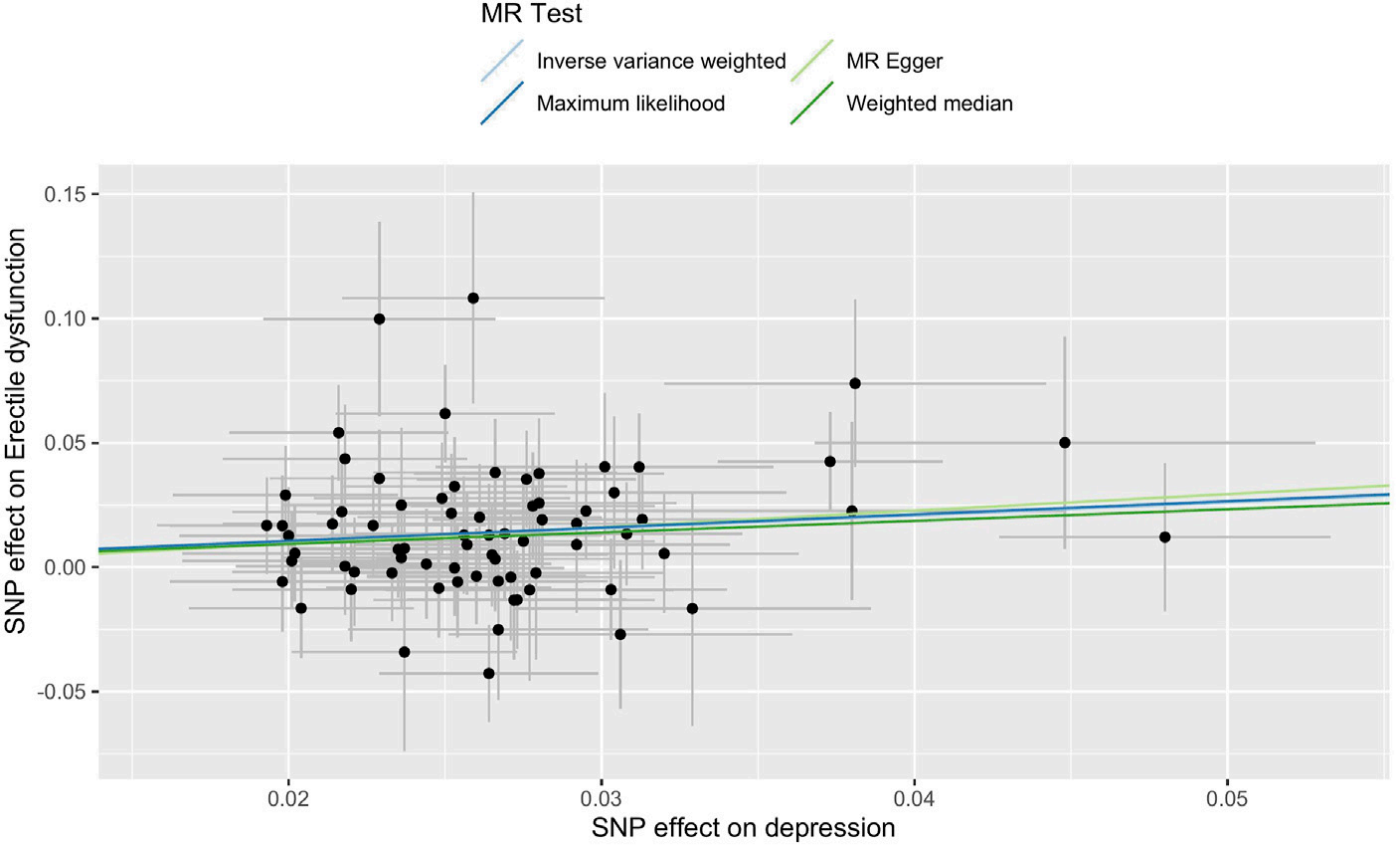
Scatter plot of the effect size of each SNP on depression and ED in MR. Abbreviations: SNP, single-nucleotide polymorphism; ED, erectile dysfunction; MR, Mendelian randomization.

**FIGURE 4:**
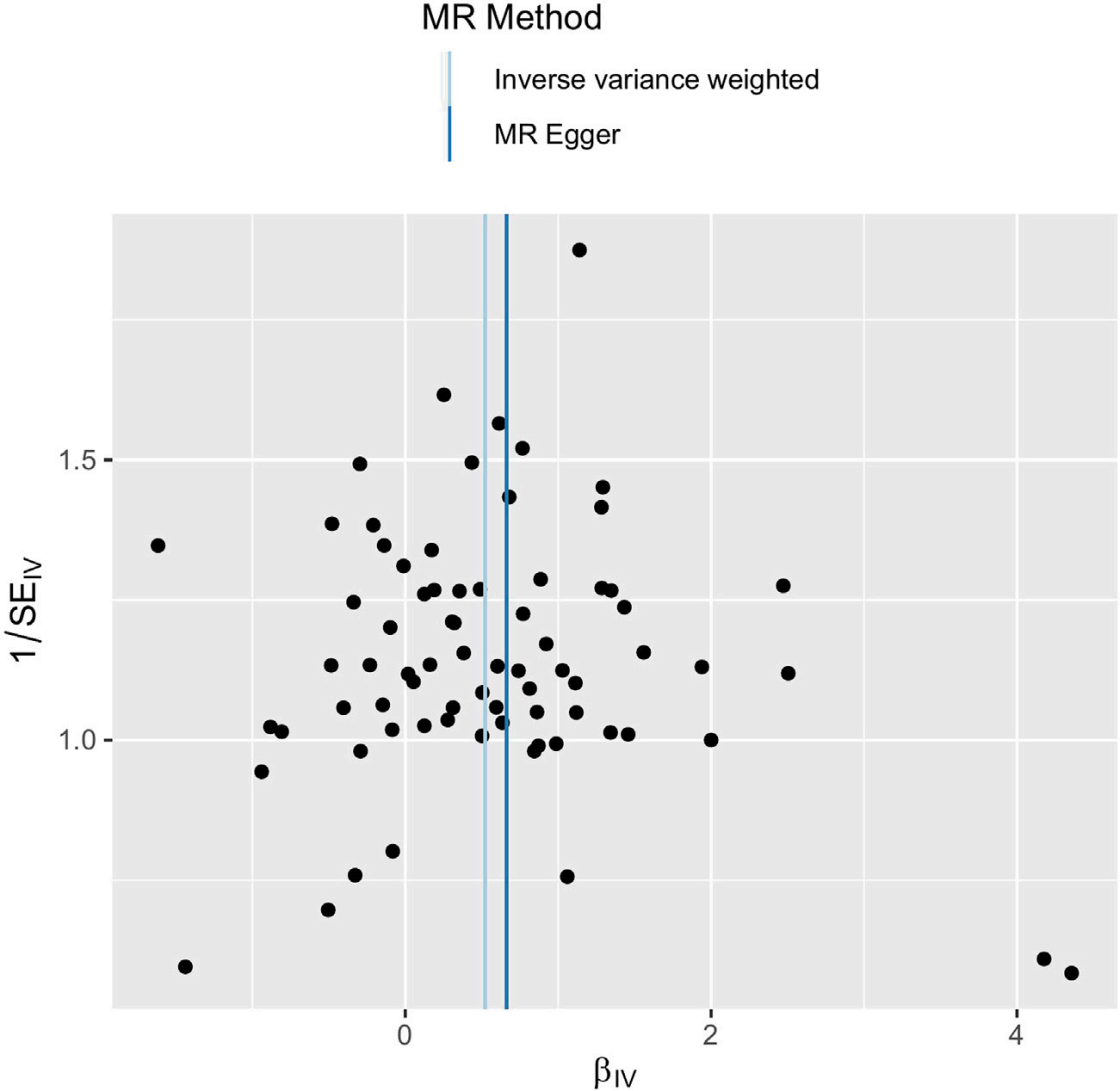
Funnel plot of SNPs used in MR of depression on ED. Abbreviations: SNP, single-nucleotide polymorphism; β, the effect size; SE, the standard error of the effect size; IVs, instrumental variables; ED, erectile dysfunction; MR, Mendelian randomization.

**FIGURE 5:**
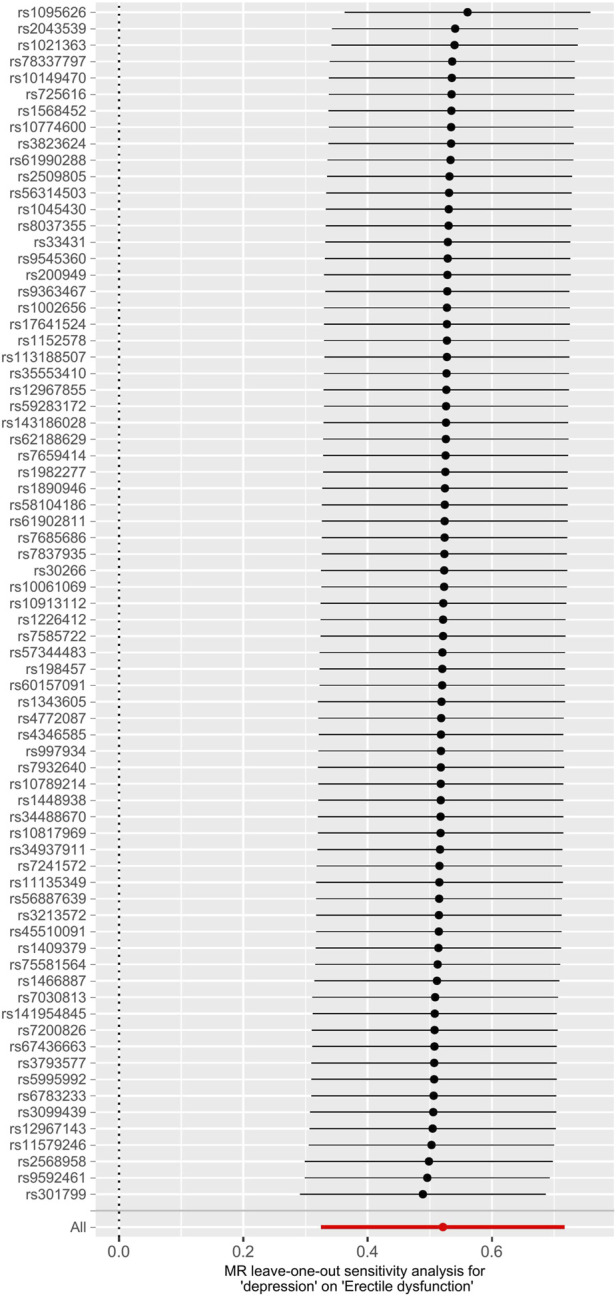
Leave-one-out analysis of depression on ED. Abbreviations: ED, erectile dysfunction; MR, Mendelian randomization.

The main causal relationship estimates of depression on ED revealed that depression could elevate the incidence of ED (IVW: OR = 1.68, 95% confidence intervals [CI]: 1.38–2.05, *p* < 0.001) (Figure 1 and Table 2). In addition, the ORs for the maximum likelihood method, MR-Egger, weighted median, MR.RAPS, and MR-PRESSO were 1.70 (95% CI = 1.39–2.08, *p* < 0 .001), 1.94 (95% CI = 0.63–6.01, *p* > 0 .05), 1.59 (95% CI = 1.21–2.10, *p* < 0 .001), 1.70 (95% CI = 1.39–2.08, *p* < 0 .001), and 1.68 (95% CI = 1.40–2.04, *p* < 0 .001), respectively (Table 2 and Table 3). The forest maps in Figure 2 indicated each SNP’s effect for depression on ED and their whole estimates. As shown in Figure 3, the risk of ED correspondingly increased as the significance of IVs on depression increased. The Cochran’s Q statistics in IVW, maximum likelihood method, and MR-Egger were 69.38 (*p* = 0.566), 68.88 (*p* = 0.582), and 69.31 (*p* = 0.534), respectively (Table 2), indicating a scanty demonstration of heterogeneity. The funnel plot displayed in Figure 4 visualized the heterogeneity. No directional pleiotropy was detected in the MR-Egger test (intercept = −0.0038, *p* = 0.804) and MR-PRESSO test (global test *p* = 0.594). No outlier SNPs were detected in MR-PRESSO analysis, suggesting limited evidence of pleiotropic bias. In addition, according to the leave-one-out analysis, no significant SNPs were driving the relationship between depression and ED (Figure 5).

**TABLE 2:** Heterogeneity test of MR analysis.

Method	Nsnp	OR (95% CI)	*p*	Q	Q_df	Q_pval
MR-Egger	73	1.94 (0.63,6.01)	0.254138	69.31	71	0.534
IVW	73	1.68 (1.38,2.05)	1.91E−07	69.38	72	0.566
Maximum likelihood	73	1.70 (1.39,2.08)	1.72E−07	68.88	72	0.582

**TABLE 3:** Pleiotropy test.

MR-PRESSO						
MR analysis	Causal estimate	sd	T-stat	*p-*value	Global.Test.RSSobs	Global.Test.Pvalue
Raw	0.5212	0.098243	5.305186	1.19E-06	71.25295	0.594
Outlier-corrected	–	–	–	–	–	–
MR-Egger						
Egger_intercept	se	pval				
−0.003757972	0.015053	0.803583				

Additionally, an online tool (https://sb452.shinyapps.io/overlap/) is used to calculate the overlap bias. The bias value is 0.001 with a type I error rate of 0.05 and a 100% overlap proportion assumption, which suggests that the demographic overlap is less likely to skew the results.

## Discussion

It was challenging to determine the causal link between depression and ED without long-term prospective research and tightly controlled randomized trials. This study offered a genetically potential causal proof that depressed patients have a greater risk of ED within the scope of the MR design.

Earlier observational studies had described the association between depression and ED (Araujo et al., 1998; Shabsigh et al., 1998; Seftel et al., 2004; Shiri et al., 2007; Nwakanma and Ofoedu, 2016). Men who had untreated depression in the historic Male Massachusetts Aging Study (MMAS) were nearly twice as likely to report ED compared to males without depression after adjusting the ages (Araujo et al., 1998). Similar findings may be derived from the Multinational Men’s Attitudes to Life Events and Sexuality research, where depressive symptoms were found in 25% of men with ED and only 13% of those without ED (Rosen et al., 2004). Nevertheless, other studies discovered no correlation between the prevalence of ED and depressive symptoms (Kantor et al., 2002; Mak et al., 2002; Wong et al., 2009; Habibi et al., 2011; Kim et al., 2015). Of the 334 patients sampled, Kantor et al. (2002) indicated that present depressed symptoms were not linked to mild or severe erectile dysfunction. In addition, psychiatrists might be hesitant to probe a patient regarding ED in depth (ClaytonMcGarvey et al., 2001).

Our study supported the previous research that depression increased the prevalence of erectile dysfunction at the genetic level. A clear causal direction could facilitate clinical decision-making. Clinicians and decision-makers should pay attention to the ED symptoms of depression patients to improve overall patient care.

Although the causal relationship between depression and ED has been studied in detail, more research into the precise molecular pathways is still required. In general, two theories existed regarding how depression causes ED: the “behavior-based” model and the “biologic-based” one. According to the behavior-based model, depressed individuals exhibited behaviors or beliefs that led to performance anxiety, which negatively impacted erectile function. Meisler and Carey (1991) showed that mood might alter sexual desire, which supported the behavior model. The biological hypothesis was well articulated by Goldstein (2000), who noted that ED and inadequate cavernosal muscle relaxation were both caused by excess catecholamine production, which was caused by depression. In addition, the dopamine system and the dopaminergic synapse signaling pathway were dysfunctional in the depression rat model (Hong et al., 2022).

This study included several strengths and limitations. The primary advantage was that the MR analysis reduced endogeneity and bias caused by confounding variables. Considering the challenge of implementing RCTs, this study offered a genetic proof that depression causes impotence. Additionally, SNP data sources were restricted to people of European individuals, which limited the applicability of our findings to other races while avoiding population structure bias. Additionally, there might be some crossover between the samples of depression and ED, which could cause the models to be overfitted and reduce the strength of causal inference (Burgess et al., 2016). Despite this, the bias might not be very noticeable based on our study’s use of robust IVs (F-statistics > 10). Additionally, the non-linear relationship between depression and ED cannot be investigated because of the binary evaluation of depression and the absence of precise individual statistics (Burgess et al., 2016). Lastly, several confounding factors may also influence the causal associations between depression and ED. There were genetically complex associations between depression and other traits, such as insomnia, cardiovascular diseases (CVDs), and neuroticism. Previous studies demonstrated that insomnia and cardiovascular diseases shared multiple genetical variants with depression, which were also the risk factors of erectile dysfunction (Zhang et al., 2021a; Baranova et al., 2022). Short sleep duration was a risk factor for CVD in spite of observational studies or MR analysis (Ai et al., 2021; Wang et al., 2022). In addition, neuroticism was genetically associated with depression and showed substantial genetic overlaps with CVD, making it a potential confounder for our study (Zhang et al., 2021b; Zhang et al., 2022).

## Conclusion

In conclusion, this study offered a genetic proof that depression may contribute to the development of ED. For more thorough and careful treatment of ED patients, medical therapies should be considered for patients with depression.

## Data Availability

Publicly available datasets were analyzed in this study. These data can be found at: https://gwas.mrcieu.ac.uk/.

## References

[B1] Ai S. Zhang J. Zhao G. Wang N. Li G. So H. C. (2021). Causal associations of short and long sleep durations with 12 cardiovascular diseases: Linear and nonlinear mendelian randomization analyses in UK Biobank. Eur. Heart J. 42, 3349–3357. 10.1093/eurheartj/ehab170 33822910

[B2] Araujo A. B. Durante R. Feldman H. A. Goldstein I. McKinlay J. B. (1998). The relationship between depressive symptoms and male erectile dysfunction: Cross-sectional results from the Massachusetts male aging study. Psychosom. Med. 60, 458–465. 10.1097/00006842-199807000-00011 9710291

[B3] Ayta I. A. McKinlay J. B. Krane R. J. (1999). The likely worldwide increase in erectile dysfunction between 1995 and 2025 and some possible policy consequences. BJU Int. 84, 50–56. 10.1046/j.1464-410x.1999.00142.x 10444124

[B4] Baranova A. Cao H. Zhang F. (2022). Shared genetic liability and causal effects between major depressive disorder and insomnia. Hum. Mol. Genet. 31, 1336–1345. 10.1093/hmg/ddab328 34761251

[B5] Bovijn J. Jackson L. Censin J. Chen C. Y. Laisk T. Laber S. (2019). GWAS identifies risk locus for erectile dysfunction and implicates hypothalamic neurobiology and diabetes in etiology. Am. J. Hum. Genet. 104, 157–163. 10.1016/j.ajhg.2018.11.004 30583798 PMC6323625

[B6] Bowden J. Davey Smith G. Burgess S. (2015). Mendelian randomization with invalid instruments: Effect estimation and bias detection through egger regression. Int. J. Epidemiol. 44, 512–525. 10.1093/ije/dyv080 26050253 PMC4469799

[B7] Bowden J. Davey Smith G. Haycock P. C. Burgess S. (2016). Consistent estimation in mendelian randomization with some invalid instruments using a weighted median estimator. Genet. Epidemiol. 40, 304–314. 10.1002/gepi.21965 27061298 PMC4849733

[B8] Brion M. J. Shakhbazov K. Visscher P. M. (2013). Calculating statistical power in Mendelian randomization studies. Int. J. Epidemiol. 42, 1497–1501. 10.1093/ije/dyt179 24159078 PMC3807619

[B9] Burgess S. Butterworth A. Thompson S. G. (2013). Mendelian randomization analysis with multiple genetic variants using summarized data. Genet. Epidemiol. 37, 658–665. 10.1002/gepi.21758 24114802 PMC4377079

[B10] Burgess S. Davies N. M. Thompson S. G. (2016). Bias due to participant overlap in two-sample Mendelian randomization. Genet. Epidemiol. 40, 597–608. 10.1002/gepi.21998 27625185 PMC5082560

[B11] Burgess S. Thompson S. G. (2011). Avoiding bias from weak instruments in Mendelian randomization studies. Int. J. Epidemiol. 40, 755–764. 10.1093/ije/dyr036 21414999

[B12] Chou P. S. Chou W. P. Chen M. C. Lai C. L. Wen Y. C. Yeh K. C. (2015). Newly diagnosed erectile dysfunction and risk of depression: A population-based 5-year follow-up study in taiwan. J. Sex. Med. 12, 804–812. 10.1111/jsm.12792 25475605

[B13] Clayton A. H. McGarvey E. L. Abouesh A. I. Pinkerton R. C. (2001). Substitution of an SSRI with bupropion sustained release following SSRI-induced sexual dysfunction. J. Clin. Psychiatry 62 (3), 185–190. 10.4088/jcp.v62n0309 11305705

[B14] Goldstein I. (2000). The mutually reinforcing triad of depressive symptoms, cardiovascular disease, and erectile dysfunction. Am. J. Cardiol. 86, 41F–45f. 10.1016/s0002-9149(00)00892-4 10899278

[B15] Habibi A. Kalbasi S. Saadatjoo S. A. Arefi M. G. (2011). Evaluation of erectile dysfunction and associated factors in type-II diabetic patients in birjand, Iran in 2008-2009. J. Res. Health Sci. 11, 97–102. 22911959

[B16] Haycock P. C. Burgess S. Wade K. H. Bowden J. Relton C. Davey Smith G. (2016). Best (but oft-forgotten) practices: The design, analysis, and interpretation of mendelian randomization studies. Am. J. Clin. Nutr. 103, 965–978. 10.3945/ajcn.115.118216 26961927 PMC4807699

[B17] Hong Z. M. Chen Z. L. Feng J. L. Wang S. J. Qiu J. F. Zeng Y. L. (2022). Mechanistic analysis of erectile dysfunction in a depression rat model. J. Int. Med. Res. 50, 030006052211003. 10.1177/03000605221100334 PMC9152200 35615771

[B18] Howard D. M. Adams M. J. Clarke T. K. Hafferty J. D. Gibson J. Shirali M. (2019). Genome-wide meta-analysis of depression identifies 102 independent variants and highlights the importance of the prefrontal brain regions. Nat. Neurosci. 22, 343–352. 10.1038/s41593-018-0326-7 30718901 PMC6522363

[B19] Kantor J. Bilker W. B. Glasser D. B. Margolis D. J. (2002). Prevalence of erectile dysfunction and active depression: An analytic cross-sectional study of general medical patients. Am. J. Epidemiol. 156, 1035–1042. 10.1093/aje/kwf142 12446260

[B20] Kim M. Kim S. Y. Rou W. S. Hwang S. W. Lee B. S. (2015). Erectile dysfunction in patients with liver disease related to chronic Hepatitis B. Clin. Mol. Hepatol. 21, 352–357. 10.3350/cmh.2015.21.4.352 26770923 PMC4712162

[B21] Laumann E. O. Kang J. H. Glasser D. B. Rosen R. C. Carson C. C. (2008). Lower urinary tract symptoms are associated with depressive symptoms in white, black and Hispanic men in the United States. J. Urol. 180, 233–240. 10.1016/j.juro.2008.03.055 18499181

[B22] Mak R. De Backer G. Kornitzer M. De Meyer J. M. (2002). Prevalence and correlates of erectile dysfunction in a population-based study in Belgium. Eur. Urol. 41, 132–138. 10.1016/s0302-2838(01)00029-x 12074399

[B23] McKinlay J. B. (2000). The worldwide prevalence and epidemiology of erectile dysfunction. Int. J. Impot. Res. 12 (4), S6–s11. 10.1038/sj.ijir.3900567 11035380

[B24] Meisler A. W. Carey M. P. (1991). Depressed affect and male sexual arousal. Arch. Sex. Behav. 20, 541–554. 10.1007/BF01550953 1768221

[B25] NIH Consensus conference (1993). Impotence. NIH Consensus development panel on impotence. Jama 270, 83–90. 8510302

[B26] Nwakanma N. C. Ofoedu J. N. (2016). Depressive symptoms and marital adjustment among primary care patients with erectile dysfunction in Umuahia, Nigeria. S. Afr. J. Psychiatr. 22, 979. 10.4102/sajpsychiatry.v22i1.979 30263170 PMC6138087

[B27] Price L. H. J. (2004). Handbook of medical psychiatry. England, UK: Routledge.

[B28] Rosen R. C. Fisher W. A. Eardley I. Niederberger C. Nadel A. Sand M. (2004). The multinational men's Attitudes to Life Events and sexuality (MALES) study: I. Prevalence of erectile dysfunction and related health concerns in the general population. Curr. Med. Res. Opin. 20, 607–617. 10.1185/030079904125003467 15171225

[B29] Salonia A. Bettocchi C. Carvalho J. Corona G. Jones T. Kadioglu A. (2020). EAU guidelines on sexual and reproductive health. AvaliableAt: https://uroweb.org/guidelines/sexual-and-reproductive-health .

[B30] Seftel A. D. Sun P. Swindle R. (2004). The prevalence of hypertension, hyperlipidemia, diabetes mellitus and depression in men with erectile dysfunction. J. Urol. 171, 2341–2345. 10.1097/01.ju.0000125198.32936.38 15126817

[B31] Seidman S. N. Roose S. P. (2000). The relationship between depression and erectile dysfunction. Curr. Psychiatry Rep. 2, 201–205. 10.1007/s11920-996-0008-0 11122956

[B32] Shabsigh R. Klein L. T. Seidman S. Kaplan S. A. Lehrhoff B. J. Ritter J. S. (1998). Increased incidence of depressive symptoms in men with erectile dysfunction. Urology 52, 848–852. 10.1016/s0090-4295(98)00292-1 9801112

[B33] Shiri R. Koskimäki J. Tammela T. L. Häkkinen J. Auvinen A. Hakama M. (2007). Bidirectional relationship between depression and erectile dysfunction. J. Urol. 177, 669–673. 10.1016/j.juro.2006.09.030 17222655

[B34] Skrivankova V. W. Richmond R. C. Woolf B. A. R. Davies N. M. Swanson S. A. VanderWeele T. J. (2021). Strengthening the reporting of observational studies in epidemiology using mendelian randomisation (STROBE-MR): Explanation and elaboration. BMJ Clin. Res. ed.) 375, n2233. 10.1136/bmj.n2233 PMC8546498 34702754

[B35] Skrivankova V. W. Richmond R. C. Woolf B. A. R. Yarmolinsky J. Davies N. M. Swanson S. A. (2021). Strengthening the reporting of observational studies in epidemiology using mendelian randomization: The STROBE-MR statement. Jama 326, 1614–1621. 10.1001/jama.2021.18236 34698778

[B36] Smith G. D. Ebrahim S. (2003). Mendelian randomization': Can genetic epidemiology contribute to understanding environmental determinants of disease? Int. J. Epidemiol. 32, 1–22. 10.1093/ije/dyg070 12689998

[B37] Steiger A. Holsboer F. Benkert O. (1993). Studies of nocturnal penile tumescence and sleep electroencephalogram in patients with major depression and in normal controls. Acta Psychiatr. Scand. 87, 358–363. 10.1111/j.1600-0447.1993.tb03387.x 8517177

[B38] Takao T. Tsujimura A. Okuda H. Yamamoto K. Fukuhara S. Matsuoka Y. (2011). Lower urinary tract symptoms and erectile dysfunction associated with depression among Japanese patients with late-onset hypogonadism symptoms. Aging Male 14, 110–114. 10.3109/13685538.2010.512374 20828247

[B39] Thase M. E. Reynolds C. F. Jennings J. R. Frank E. Garamoni G. L. Nofzinger E. A. (1992). Diminished nocturnal penile tumescence in depression: A replication study. Biol. Psychiatry 31, 1136–1142. 10.1016/0006-3223(92)90158-v 1525277

[B40] Thase M. E. Reynolds C. F. Jennings J. R. Frank E. Howell J. R. Houck P. R. (1988). Nocturnal penile tumescence is diminished in depressed men. Biol. Psychiatry 24, 33–46. 10.1016/0006-3223(88)90119-9 3370276

[B41] Verbanck M. Chen C. Y. Neale B. Do R. (2018). Detection of widespread horizontal pleiotropy in causal relationships inferred from Mendelian randomization between complex traits and diseases. Nat. Genet. 50, 693–698. 10.1038/s41588-018-0099-7 29686387 PMC6083837

[B42] Wald A. (1940). The fitting of straight lines if both variables are subject to error. J. Ann. Math. Stat. 11, 284–300.

[B43] Wang S. Li Z. Wang X. Guo S. Sun Y. Li G. (2022). Associations between sleep duration and cardiovascular diseases: A meta-review and meta-analysis of observational and mendelian randomization studies. Front. Cardiovasc. Med. 9, 930000. 10.3389/fcvm.2022.930000 36035915 PMC9403140

[B44] Wong S. Y. Leung J. C. Woo J. (2009). Sexual activity, erectile dysfunction and their correlates among 1, 566 older Chinese men in Southern China. J. Sex. Med. 6, 74–80. 10.1111/j.1743-6109.2008.01034.x 19170839

[B45] Zhang F. Baranova A. Zhou C. Cao H. Chen J. Zhang X. (2021). Causal influences of neuroticism on mental health and cardiovascular disease. Hum. Genet. 140, 1267–1281. 10.1007/s00439-021-02288-x 33973063

[B46] Zhang F. Cao H. Baranova A. (2022). Genetic variation mediating neuroticism's influence on cardiovascular diseases. J. Psychopathol. Clin. Sci. 131, 278–286. 10.1037/abn0000744 35230853

[B47] Zhang F. Cao H. Baranova A. (2021). Shared genetic liability and causal associations between major depressive disorder and cardiovascular diseases. Front. Cardiovasc. Med. 8, 735136. 10.3389/fcvm.2021.735136 34859065 PMC8631916

[B48] Zhao Q. Chen Y. Wang J. Small D. S. (2019). Powerful three-sample genome-wide design and robust statistical inference in summary-data Mendelian randomization. Int. J. Epidemiol. 48, 1478–1492. 10.1093/ije/dyz142 31298269

